# Familial Aggregation of Metabolic Syndrome Indicators in Portuguese Families

**DOI:** 10.1155/2013/314823

**Published:** 2013-09-22

**Authors:** D. M. Santos, P. T. Katzmarzyk, D.-A. Trégouet, T. N. Gomes, F. K. Santos, J. A. Maia

**Affiliations:** ^1^CIFI^2^D, Faculty of Sports, University of Porto, Rua Dr. Plácido Costa 91, 4200-450 Porto, Portugal; ^2^Pennington Biomedical Research Center, Louisiana State University System, 6400 Perkins Road, Baton Rouge, LA 70808, USA; ^3^INSERM, UMR S937, Institute for Cardiometabolism and Nutrition (ICAN), Pierre and Marie Curie University (UPMC, Paris6), 75654 Paris Cedex 13, France

## Abstract

*Background and Aims*. Family studies are well suited to investigate the genetic architecture underlying the metabolic syndrome (MetS). The purposes of this paper were (i) to estimate heritabilities for each of the MetS indicators, and (ii) to test the significance of familial intratrait and cross-trait correlations in MetS markers. *Methods and Results*. This study included 1,363 individuals from 515 Portuguese families in which five MetS components, including waist circumference (WC), blood pressure (BP), HDL-cholesterol, triglycerides (TG), and glucose (GLU), were measured. Intratrait and cross-trait familial correlations of these five components were estimated using Generalized Estimating Equations. Each MetS component was significantly heritable (*h* ^2^ ranged from 0.12 to 0.60) and exhibited strong familial resemblance with correlations between biological relatives of similar magnitude to those observed between spouses. With respect to cross-trait correlations, familial resemblance was very weak except for the HDL-TG pair. *Conclusions*. The present findings confirm the idea of familial aggregation in MetS traits. Spousal correlations were, in general, of the same magnitude as the biological relatives' correlations suggesting that most of the phenotypic variance in MetS traits could be explained by shared environment.

## 1. Introduction

Metabolic syndrome (MetS) is a cluster of interrelated cardiovascular disease risk factors characterized by glucose intolerance, hypertension, dyslipidemia, and obesity and is associated with increased risk of cardiovascular disease mortality in adults [1–3]. The mechanisms underlying the MetS physiopathogenesis are complex, both genetic and environmentally driven, and factors like physical inactivity and poor dietary habits are likely to condition the emergence and further development of cardiometabolic disorders [4].

MetS prevalence has been increasing in the last decades, namely, in the western, more modernized countries [5]. However, available data is far from being concordant as different definitions and cut-points are being used to determine risk [6].Using NCEP-ATPIII criteria, a large hospital Portuguese sample of adults (age range: 18–96 y) showed a MetS prevalence of 27.5% [7] which is compared to 34% in the USA [8].

Studying families may be of great assistance in unraveling the importance of shared genes and environmental conditions since common behaviors within family lifestyles may trigger the appearance of MetS [9]. The first step to assess the genetic contribution to a trait is to calculate its familiality and to derive heritability (*h* ^2^) estimates [10]. For example, in a recent report from Vattikuti et al. [9], *h* ^2^ ranged from 0.30 to 0.48 in MetS traits. Also, in a study by Tang et al. [11] *h* ^2^ were of the same magnitude, varying from 0.33 (DBP) to 0.63 (HDL). Moreover, in the NHLBI sample [11], significant familial intratrait correlations were observed between parents and offspring for diastolic blood pressure (DBP), HDL-cholesterol (HDL), and fasting insulin (INS), supporting the hypotheses of shared effects (genetic and/or environmental) on these MetS indicators. 

Bivariate familial correlation analysis can be of great help to explore the possibility of shared genetic and/or environmental effects among MetS traits. In this case, genetic correlation coefficients provide information on the amount of additive genetic variation shared between two MetS traits. This could be of great importance in understanding if two traits are more closely related than others. Potentially, this could influence the development of intervention programs. For instance, Trégouet et al. [12] found that body mass index (BMI)/insulin (INS) correlations between biological relatives tend to be higher than those between spouses, supporting the hypotheses of common transmissible factors for these two traits. Also, using a twin design with 405 MZ pairs and 290 DZ pairs, Pang et al. [13] found moderate to high correlations between total cholesterol (TC) and LDL-cholesterol (LDL) (*r* = 0.84), LDL and HDL (*r* = 0.62), TC and HDL (*r* = 0.58), triacylglycerol (TRG) and TC (*r* = 0.45), and TG and LDL (*r* = 0.41). In a recent review, Povel et al. [14] verified that genetic correlation coefficients were strongest for waist circumference and HOMA-IR (median *r* = 0.59) and for HDL and TG (median *r* = 0.46). On the opposite side, the genetic correlation coefficients of BP and HDL (median *r* = 0.09), BP and TG (median *r* = 0.10), BP and fasting glucose (median *r* = 0.13), and fasting glucose and HDL (median *r* = 0.07) were the lowest. Vattikuti et al. [9] found significant genetic correlations between GLU-INS (*ρ*_*G*_ = 0.69), INS-TG (*ρ*_*G*_ = 0.76), and TG-HDL (*ρ*_*G*_ = −0.59) in the Aric population.

The present report pursues two specific aims: (i) to estimate heritabilities (*h* ^2^) for each of the MetS indicators and (ii) to test the significance of familial intratrait and cross-trait correlations in MetS markers.

## 2. Materials and Methods

### 2.1. Study Population

*The Healthy Family study*, from the Portuguese *Famílias Saudáveis* (FAMS), investigates the relationship among MetS traits, physical activity, physical fitness, and body composition in families. Children and adolescents aged ≤18 years were recruited in schools from Azores and Madeira archipelagos, and north and central regions of mainland Portugal, and were approached to freely participate in the study with their siblings and parents. School officers provided family lists, andfamilies with at least two siblings were initially invited. However, given that families with 3 or more children are scarce in the Portuguese population [15], and to improve statistical power, we also invited one-offspring families through random eligibility. Children with chronic diseases, physical handicaps, or psychological disorders were excluded as these conditions might impair their daily routines, namely, their physical activities within schools and/or sports clubs. As such, our sample comprises 515 families with one or two offsprings (see Table 1). Some relatives were not able to participate in the study and the resulting sample was 252 fathers, 464 mothers, 317 sons, and 330 daughters. The Ethics Committee of the Faculty of Sport, University of Porto, approved the study, and written informed consent was obtained from all subjects.

### 2.2. Data Collection

The standardized procedures of Lohman et al. [16] were used to measure height with a Siber Hegner anthropometer (GMP instruments) and weight with a Tanita scale (model BC-418 MA); waist circumference was measured at the end of a normal expiration just above the iliac crest, using a nonelastic Holtain tape. 

Blood samples were collected after an overnight fast of at least 10 to 12 h. Glucose, TC, HDL, and TG were analyzed with an LDX point of care analyzer [17]. This method has been previously validated against a laboratory reference method [18], and daily optical equipment checks were made according to manufacturer instructions.

Resting systolic (SBP) and diastolic (DBP) blood pressures were measured with an Omron Model M6 (HEM-7001-E) device according to the International Protocol of the European Society of Hypertension [19]. Cuff sizes were modified depending on the size of the subject's arm. Subjects were seated in an upright position with the right arm resting on a table at the heart level. The first reading was performed after a 5-minute resting period. The other two readings were performed with three minute breaks in between. The mean of the three blood pressure measurements was used for analysis.

### 2.3. Statistical Analysis

Descriptive continuous data are presented as means ± SDs, whereas dichotomous data are presented as percentages.

Heritability estimates were computed using a maximum likelihood approach implemented in SOLAR v.4.3.1 software [20]. In order to handle the nonindependence of the family observations, the intratrait and cross-trait familial correlation analyses were conducted within the framework of Generalized Estimating Equations (GEEs), an elegant and efficient alternative to maximum likelihood methods that does not require any distributional assumptions. GEEs are highly flexible for studying covariate effects on means and correlations and are asymptotically robust to a misspecification of the exact pattern of correlations between observations. GEEs are in particular well-suited to family data analysis where observations between relatives may be correlated due to shared environment and genetic factors [21]. For the current application, we used the GESEE and BIEE software developed by Trégouet et al. [12] as previously applied by Plancoulaine et al. [22–24]. 

According to the given family structure, up to eight different types of family intratrait correlations could be estimated. For this particular study we decided to focus on *ρ*_FM_ = father-mother, *ρ*_FO_ = father-offspring, *ρ*_MO_ = mother-offspring, and *ρ*_SS_ = sibling-sibling. The generalized Wald test statistic was then employed to test specific hypotheses on these correlations. For example, we were particularly interested in testing whether (1) all four correlations were equal to zero, meaning that there is no familial aggregation of MetS traits, (2) all correlations were equal to each other, which would suggest a strong shared environmental component, or (3) *ρ*_FO_ = *ρ*_MO_ = *ρ*_SS_ suggesting that no sex differences exist in terms of parental transmission of MetS traits.

For the cross-trait analyses, two different types of correlations were studied: cross-trait within individual correlations per class of relatives (e.g., correlation between glucose and SBP in fathers) and cross-trait interindividual correlations (e.g., correlation between glucose levels in a father and SBP in his offspring). For the latter, we assumed a symmetric correlation pattern for each pair of traits, with four cross-trait interindividuals correlations according to classes of relatives *ρ*_FM_ = spouses, *ρ*_FO_ = father-offspring, *ρ*_MO_ = mother-offspring, and *ρ*_SS_ = sibling-sibling. This implies, for example, that the correlation between glucose in a father and SBP in his offspring is the same as the correlation between the SBP in the father and the glucose in his offspring.

Strong cross-trait correlations between biological relatives but not between spouses would suggest the existence of shared genetic factors influencing both traits. Additionally, observing cross-trait correlations between spouses would suggest shared environmental factors.

In all analyses, phenotypic means were adjusted for age, age^2^, sex, age × sex, and age^2^ × sex. The level of statistical significance was set at 0.05.

## 3. Results

Table 1 presents the descriptive data for the sample. A total of 1,363 subjects from 515 families were included. The average family size was 2.6 subjects. Correlations were derived from the following family combinations: 240 spousal, 216 father-offspring, 283 mother-offspring, and 208 sibling-sibling pairs.

All traits were found to be highly heritable (Table 2) with estimates ranging from 0.12 (GLU) to 0.60 (WC). This means that there are strong additive genetic effects on the expression of these MetS trait that justifies further specific analysis of their genetic architecture.

Table 3 shows the familial correlations and their 95% confidence intervals for each MetS trait. The absence of familial resemblance was rejected for each phenotype (all *P* < 10^−4^), and different general family correlations were observed.

For GLU, SBP, and TG, the test for equality of all correlations was not rejected, *P* = 0.24, *P* = 0.21, and *P* = 0.23, respectively. The resulting common familial correlations were GLU, *ρ* = 0.30 (95% confidence interval: [0.19–0.40]), SBP, *ρ* = 0.26 [0.19–0.32]), and TG, *ρ* = 0.16 [0.08–0.23]. For HDL (*P* < 10^−4^) and WC (*P* < 10^−4^), the test for equality of all correlations was significant. Furthermore, for HDL the biological relatives correlations were significantly different (*P* = 0.01) from each other, with the lowest correlation of 0.28 for father-offspring and the highest of 0.49 for sib-sib. For WC no significant differences were observed between genetic related relatives (*P* = 0.327), but mother-offspring correlation (*ρ* = 0.45 [0.34–0.55]) was higher than both father-offspring (*ρ* = 0.30 [0.25–0.50]) and sib-sib (*ρ* = 0.30 [0.18–0.42]) correlations. HDL and TG did not exhibit significant spouse correlation suggesting a low environment component for these phenotypes. However, TG correlations were also nonsignificant for father-offspring and sib-sib correlations.

Cross-trait within-individuals correlations are shown in Table 4. The pairs of traits that demonstrated significant family correlations were GLU-WC (*P* < 0.001), HDL-SBP (*P* = 0.039), and HDL-TG (*P* < 0.01). Moreover it can be observed that glucose was poorly correlated with other MetS traits in a given individual, except with TG and WC in fathers only. Second, HDL was negatively correlated with TG in all class of relatives and with SBP in sons. Furthermore, SBP was positively correlated with daughters' TG and parents' WC. Lastly, TG positively correlated with WC in male family members.

As for the cross-trait familial correlations computed for the four familial dyads (Table 5), results showed that within GLU-SBP (*P* < 0.001), GLU-WC (*P* = 0.027), GLU-TG (*P* = 0.049), HDL-WC (*P* = 0.010), SBP-WC (*P* = 0.017), and SBP-TG (*P* = 0.046), the specific familial correlations are significantly different from each other. From these, sibling correlations were only significant for GLU-WC (*ρ* = −0.10) and HDL-WC (*ρ* = 0.14). On the other hand, spouses correlations were significant for SBP-GLU (*ρ* = −0.12) and SBP-WC (*ρ* = 0.13). Lastly, father-offspring correlations were significant for GLU-TG (*ρ* = 0.08), and mother-offspring correlations were significant for HDL-WC (*ρ* = 0.08).

## 4. Discussion

In this study detailed information about MetS in a sample of 515 Portuguese families is presented. We analyzed the complex network of interrelationships between each trait within a family.

The present findings confirm that MetS traits are highly heritable in agreement with previous results [9, 11]. For instance, in the present study, the heritability estimate for HDL was 0.44, which is close to previous estimates given by Vattikuti et al. [9] and by the NHLBI [11] study, 0.48 and 0.63, respectively. Furthermore, physical exercise, as well as statins, have been shown to induce positive changes in HDL concentrations, via direct or mediated influences [25, 26]. WC (*h* ^2^ = 0.60) and SBP (*h* ^2^ = 0.50) genetic factors were slightly higher in our study than in the NHLBI sample, which amounted to 0.42 and 0.33, respectively. Again, physical exercise and nutrition are well-known for their strong and positive impact on WC and SBP traits suggesting that shared environment will probably be responsible for a great amount of the phenotypic variance in our sample. As for GLU and TG, the present results for heritability are lower when compared to those of other findings [9, 11] with *h* ^2^ ranging from 0.12 to 0.29. In any case, we have to emphasize that heritability estimates are also influenced by familial environment correlates, since no control was made for the possible effects of shared household characteristics during childhood and adulthood.

Familial intratrait correlations of MetS components were estimated, and familial similarity was found for each trait (all *P* < 10^−4^) even though different magnitudes were established. Correlation equality was found in SBP, GLU, and TG and between each biological family dyad in WC. Using the same statistical approach, Trégouet et al. [12] found that spouse correlations were always different from zero, meaning that there is a possibility of a shared environmental factor influencing MetS traits. Furthermore, the Johnson et al. [27] results using a French sample also highlighted that the correlations between biological relatives were always greater than those between spouses for the five MetS traits. This was not the case in our sample where, besides HDL, spouse correlations were of the same magnitude as for the biological relatives' correlations. Spousal resemblance may be attributed to distinct processes, namely, marital interaction (increasing mutual influence process across time of marriage), social homogamy (incidental resemblance due to some cultural and/or social background), and phenotypic assortment (selection process due to some characteristic) [28]. It has been suggested that assortative mating by BMI was associated with increasing prevalence of obesity in offspring in several developed populations [29, 30]. If this is true, one could speculate that phenotypic correlations in MetS traits between spouses might be a function of assortative mating, although no studies have demonstrated, so far, this assumption. Also, it has been hypothesized that phenotypic variation is associated with the duration of the marriage as an indicator of social interaction [31]. Unfortunately, no adjustment was made for marriage years, but it is possible that the spousal correlations may reflect marital interaction processes since both spouses share many cultural assets, health beliefs, social background, and experiences as couples. Moreover, controlling for other lifestyle/behavioral variables, like impaired fetal growth that has been associated with insulin resistance and other MetS traits [32], would probably lead to a better understanding of the correlations. In this case, adjusting for life history variables during pregnancy would probably add information on siblings' correlations because of their exposure to possible different environments in utero. Other lifestyle variables have been used to limit to a minimum the influence of familial environment, but even when such adjustments were made, residual effects were still identified as capable of influencing the results [11]. This ensemble of results supports a very strong argument for the existence of familial influence on MetS traits, due to additive and/or interactive genetic and shared environmental factors. 

Lastly, cross-trait correlations for all MetS traits were computed. This is very relevant in helping disentangle the genetic and environmental mechanisms that may rule the association between traits. We took two different approaches: (i) analyzing each family relative and (ii) analyzing four different family dyads. Correlation in each relative was always significant and negative for HDL/TG. This result is in agreement with previous findings [14] in which low HDL is a result of inefficient catabolism of TG rich lipoproteins and reduced transfer of surface components to nascent HDL particles [33]. Moreover, depletion of cholesterol HDL particles is also favored in the presence of TG-enriched HDL particles that are more rapidly catabolized by lipases [34]. Recently, a set of genetic variants in the LPL gene and in the APOA1/APOC3/APOA4/APOA5 gene cluster was found to mediate the significant genetic correlation between TG and HDL [35]. However, our results fail to replicate some previous reports [11, 36–38], but it needs to be recognized that the vast majority of the studies did not only use different statistical methods and did not present information on the four dyads. For instance, we could not reproduce the results of Butte et al. [36] in which environmental correlations of MetS traits were all significant, with the exceptions of SBP/GLU and TG/GLU, and genetic correlations were significantly different from 1 (i.e., partial pleiotropy), meaning that the environment also plays a role on determining the relationship between the traits. However, it is undisputable that pleiotropy among some MetS components is present and it has been proven by familial studies [37, 39] as well as by genome scans [40, 41]. Such incongruities may also be linked to population characteristics and/or covariates adjustment, but the hard fact is that actual knowledge is not conclusive about the biological mechanisms that regulate the association between some MetS traits [11]. And, in such cases in which there is evidence for shared physiology pathways, like in GLU and TG [42], genetic analysis as the present report fail to consubstantiate that datum.

The cross-trait correlations between dyads yielded a different insight into the relationship between relatives and traits. In the present sample, WC correlated significantly with all of the other traits, at least in one dyad, meaning that body fat of a relative could be associated with other traits in another relative. On the other hand, in the Trégouet et al. [12] study, the marker for obesity was BMI, and it was not significantly correlated with TG. As TG is clearly related with body fat, it seems that environmental factors may play a key role in this association within a family, as nutritional habits, and a sedentary lifestyle have been found to aggregate in families. Interestingly, we found a very similar number of significant correlations for each dyad, even though they involved different traits. It seems clear from these results that MetS is truly a cluster of nested traits that are dependent on each other, whatever the nature of dependency: genetic or environmental.

Some limitations of the present study need to be addressed. Firstly, because our sample depends on the bias associated with a free health check-up, it may not be representative of the general Portuguese population. Moreover, it is possible that these families are in a healthier condition than the overall population, making it harder to detect clusters of cardiovascular risk factors in comparison to high-risk samples. Also, the possibility of false positive results due to multiple statistical testing should not be ruled out. Furthermore, we were not able to account for biological maturation in adolescents which could influence our results. Finally, the absence of data on insulin levels may limit the understanding of some traits' relationships.

This study has several strengths such as the large sample size and the reliance on continuous data for MetS traits, as well as the use of statistical methods such as GEE which allowed us to tackle the dependence among family members.

## 5. Conclusions

In summary, the present findings confirm the idea of familial aggregation in MetS traits. However, the spousal correlations were of the same magnitude as the biological relatives correlations (with the exception of HDL) impelling us to suggest that shared environment is of utmost importance on the phenotypic expression of MetS traits. Genetic pleiotropy might exist between HDL and TG. Future research on genetic variants mediating MetS traits' correlations would lead to a better understanding of MetS etiology. 

## Figures and Tables

**Table 1:** Sample descriptive characteristics (means ± standard deviations).

	Fathers (*n* = 252)	Mothers (*n* = 464)	Sons (*n* = 317)	Daughters (*n* = 330)
Age (yrs)	46.8 ± 8.9	43.8 ± 8.6	14.0 ± 6.2	14.9 ± 8.3
Height (cm)	169.9 ± 6.6	158.1 ± 6.3	157.2 ± 16.3	152.8 ± 12.5
Weight (kg)	81.8 ± 13.2	68.6 ± 12.4	55.9 ± 17.8	53.1 ± 15.1
BMI (kg/m^2^)	28.3 ± 4.1	27.5 ± 4.7	22.1 ± 4.3	22.4 ± 4.6
WC (cm)	95.8 ± 10.8	86.2 ± 11.6	74.7 ± 12.1	73.3 ± 13.5
SBP (mmHg)	133.7 ± 17.3	124.9 ± 16.5	116.0 ± 13.9	113.9 ± 12.7
DBP (mmHg)	81.64 ± 10.62	77.37 ± 10.93	65.38 ± 9.51	67.62 ± 9.00
HDL (mg/dL)	46.4 ± 15.1	55.8 ± 14.6	49.5 ± 14.9	52.8 ± 13.6
TG (mmol/L)	1.74 ± 1.11	1.28 ± 0.68	0.78 ± 0.41	0.92 ± 0.55
Glucose (mg/dL)	97.5 ± 19.1	86.2 ± 11.7	83.8 ± 8.7	82.7 ± 8.6

BMI: body mass index; WC: waist circumference; SBP: systolic blood pressure; DBP: diastolic blood pressure; HDL: high density lipoprotein; TG: triglycerides.

**Table 2:** Heritability (*h* ^2^) estimates of the different phenotypes in the healthy families study.

Trait	*h* ^2^	Std. error	*P* value	CI_95%_
WC	0.60	0.05	<0.001	0.52–0.67
SBP	0.50	0.05	<0.001	0.42–0.59
GLU	0.12	0.05	<0.001	0.03–0.20
HDL	0.44	0.05	<0.001	0.36–0.52
TG	0.29	0.06	<0.001	0.20–0.38

WC: waist circumference; SBP: systolic blood pressure; GLU: glucose; HDL: high density lipoprotein; TG: triglycerides.

**Table 3:** Intra-trait familial correlations of MetS traits and corresponding 95% confidence intervals.

Trait	*ρ*_FM_ = 0.07	*ρ*_FO_ = 0.07	*ρ*_MO_ = 0.07	*ρ*_SIBS_ = 0.07	All correlations are equal to zero	All correlations are equal	Equality between biological relatives
GLU	0.39 (0.18–0.57)	0.29 (0.15–0.43)	0.29 (0.17–0.41)	0.27 (0.13–0.39)	*P* < 0.0001	*P* = 0.236	
SBP	0.23 (0.09–0.37)	0.20 (0.09–0.31)	0.27 (0.18–0.36)	0.31 (0.17–0.44)	*P* < 0.0001	*P* = 0.211	
HDL	0.10 (−0.03–0.24)	0.28 (0.14–0.40)	0.39 (0.25–0.52)	0.49 (0.27–0.66)	*P* < 0.0001	*P* < 0.001	*P* = 0.015
TG	0.15 (−0.01–0.31)	0.13 (−0.01–0.26)	0.22 (0.08–0.34)	0.12 (0.00–0.23)	*P* < 0.0001	*P* = 0.234	
WC	0.38 (0.25–0.50)	0.30 (0.19–0.41)	0.45 (0.34–0.55)	0.30 (0.18–0.42)	*P* < 0.001	*P* = 0.021	*P* = 0.327

HDL: high density lipoprotein; GLU: glucose; SBP: systolic blood pressure; TG: triglycerides; WC: waist circumference.

**Table 4:** Cross-trait correlations of MetS components within individuals according to each class of relatives.

Trait	HDL	SBP	TG	WC
GLU	*ρ*_F_ = −0.01	0.08	0.13*	0.20*
	*ρ*_M_ = −0.02	−0.01	0.03	0.08
	*ρ*_S_ = −0.01	−0.07	0.02	−0.12
	*ρ*_D_ = −0.09	0.02	0.10	−0.08

HDL		−0.06	−0.17*	−0.04
		0.03	−0.11*	−0.06
		−0.15*	−0.12*	0.05
		−0.05	−0.13*	0.04

SBP			0.07	0.12*
			0.04	0.05
			−0.00	−0.00
			0.10*	−0.07

TG				0.11*
				0.04
				0.13*
				0.08

*Correlations that are significantly different from 0 at *P* < 0.05.

HDL: high density lipoprotein; GLU: glucose; SBP: systolic blood pressure; TG: triglycerides; WC: waist circumference.

Each pair of traits is characterized by 4 types of within-individual correlations in fathers (*ρ*_F_), in mothers (*ρ*_M_), in sons (*ρ*_S_), and in daughters (*ρ*_D_).

**Table 5:** Cross-trait interindividuals correlations of MetS components according to each class of relatives.

Trait	HDL	SBP	TG	WC
GLU	*ρ*_FM_ = 0.07	−0.12*	0.04	0.08
	*ρ*_FO_ = 0.06	0.01	0.08*	0.04
	*ρ*_MO_ = 0.04	−0.12*	0.06	−0.02
	*ρ*_SIBS_ = 0.04	−0.08	0.05	−0.10*

HDL		−0.08	−0.05	0.09
		0.08	−0.07	0.07
		−0.08	−0.08*	0.08*
		−0.09	−0.08	0.14*

SBP			0.07	0.13*
			0.01	0.05
			0.01	−0.00
			0.01	−0.07

TG				0.03
				0.08*
				0.07*
				0.07

*Significantly different from 0 at *P* < 0.05.

HDL: high density lipoprotein; GLU: glucose; SBP: systolic blood pressure; TG: triglycerides; WC: waist circumference.

Each pair of traits is characterized by 4 types of inter-individual correlations: between spouses (*ρ*_FM_), father-offspring (*ρ*_FO_), mother-offspring (*ρ*_MO_), and sib-sib (*ρ*_SS_).

## Acronyms

GLU: Glucose
TG: Triglycerides
HDL: High density lipoprotein cholesterol
SBP: Systolic blood pressure
WC: Waist circumference
TRG: Triacylglycerol
TC: Total cholesterol
MetS: Metabolic syndrome
BP: Blood pressure
INS: Insulin
BMI: Body mass index
DBP: Diastolic blood pressure
LDL: Low density lipoprotein.
